# Evaluation of the effect of melatonin in patients with COVID-19-induced pneumonia admitted to the Intensive Care Unit: A structured summary of a study protocol for a randomized controlled trial

**DOI:** 10.1186/s13063-021-05162-3

**Published:** 2021-03-08

**Authors:** Ali Ameri, Masoomeh Frouz Asadi, Manoochehr Kamali, Majid Vatankhah, Ava Ziaei, Omid Safa, Masoomeh Mahmudi, Mohammad Fathalipour

**Affiliations:** 1 Student Research Committee, Hormozgan University of Medical Sciences, Bandar Abbas, Iran; 2 Anesthesiology, Critical Care, and Pain Management Research Center, Hormozgan University of Medical Sciences, Bandar Abbas, Iran; 3 Infectious and Tropical Diseases Research Center, Hormozgan Health Institute, Hormozgan University of Medical Sciences, Bandar Abbas, Iran; 4 Department of Clinical Pharmacy, Faculty of Pharmacy, Hormozgan University of Medical Sciences, Bandar Abbas, Iran; 5 Department of Pharmacology and Toxicology, Faculty of Pharmacy, Hormozgan University of Medical Sciences, Bandar Abbas, Iran

**Keywords:** COVID-19, Randomized controlled trial, Protocol, Melatonin, Inflammation, Intensive Care Unit

## Abstract

**Objectives:**

We investigate the effects of melatonin, compared to the usual therapeutic regimen on clinical symptoms and laboratory signs in severely ill patients with confirmed COVID-19 who are admitted to the Intensive Care Unit (ICU).

**Trial design:**

This is a single-center, open-label, randomized, clinical trial with a parallel-group design. This study is being conducted at Shahid Mohammadi Hospital, Bandar Abbas, Iran.

**Participants:**

All patients admitted to the ICU of Shahid Mohammadi Hospital, Bandar Abbas, Iran, will be screened for the following criteria.

*Inclusion criteria*

1. Age >20 years

2. Definitive diagnosis of COVID-19 based on RT-PCR or/and serological testing

3. Severe pneumonia and lung involvement in imaging

4. Signing informed consent

*Exclusion criteria*

1. Underlying diseases, including convulsive disorders, chronic hepatic and renal diseases

2. Use of mechanical ventilation

3. History of known allergy to Melatonin

4. Pregnancy and breastfeeding

**Intervention and Comparator:**

*Intervention group:* The standard treatment regimen for COVID-19, according to the Iranian Ministry of Health and Medical Education’s protocol, along with Melatonin soft gelatin capsule (Danna Pharmaceutical Company) at a dose of 5 mg twice a day for a period of seven days.

*Control group:* The standard treatment for COVID-19 based on the Iranian Ministry of Health and Medical Education’s protocol for a period of seven days.

**Main outcomes:**

The primary outcomes are the recovery rate of clinical symptoms and checking arterial blood gas (ABG), C-reactive protein (C-RP), Ferritin, Lactate dehydrogenase (LDH) within seven days of randomization.

The secondary outcomes are time to improvement of clinical and paraclinical features and length of stay in the ICU, need for mechanical ventilation, and mortality rate within seven days of randomization.

**Randomization:**

Included patients will be allocated to one of the study arms using block randomization in a 1:1 ratio (each block consists of 6 patients). This randomization method ensures a balanced allocation between the arms during the study. A web-based system will generate random numbers for the allocation sequence and concealment of participants. Each number relates to one of the study arms.

**Blinding (masking):**

This is an open-label trial without blinding and placebo control.

**Numbers to be randomized (sample size):**

A total of 60 participants randomizes (30 patients allocated to the intervention group and 30 patients allocated to the control group).

**Trial Status:**

The protocol is Version 1.0, February 16, 2021. Recruitment began February 28, 2021, and is anticipated to be completed by July 31, 2021.

**Trial registration:**

The trial protocol has been registered in the Iranian Registry of Clinical Trials (IRCT). The registration number is “IRCT20200506047323N7”. The registration date was February 16, 2021.

**Full protocol:**

The full protocol is attached as an additional file, accessible from the Trials website (Additional file 1). In the interest of expediting the dissemination of this material, the familiar formatting has been eliminated; this Letter serves as a summary of the key elements of the full protocol.

**Supplementary Information:**

The online version contains supplementary material available at 10.1186/s13063-021-05162-3.

## Supplementary Information

**Additional file 1.**

## Data Availability

The corresponding author has access to the final dataset of the trial, and the data will be available on reasonable request (Contact: M.fathalipour@hums.ac.ir).

